# Tripartite Loops Reverse Antibiotic Resistance

**DOI:** 10.1093/molbev/msaf115

**Published:** 2025-06-06

**Authors:** Farhan R Chowdhury, Brandon L Findlay

**Affiliations:** Department of Biology, Concordia University, Montréal, Québec H4B 1R6, Canada; Department of Biology, Concordia University, Montréal, Québec H4B 1R6, Canada; Department of Chemistry and Biochemistry, Concordia University, Montréal, Québec H4B 1R6, Canada

**Keywords:** tripartite loops, sequential antibiotic therapy, cyclic therapy, collateral sensitivity, epistasis, soft agar gradient evolution

## Abstract

Antibiotic resistance threatens to undo many of the advancements of modern medicine. A slow antibiotic development pipeline makes it impossible to outpace bacterial evolution, making alternative strategies essential to combat resistance. In this study, we introduce cyclic antibiotic regimens composed of 3 drugs or “tripartite loops” to contain resistance within a closed drug cycle. Through 424 discrete adaptive laboratory evolution experiments we show that as bacteria sequentially evolve resistance to the drugs in a loop, they continually trade their past resistance for fitness gains, reverting back to sensitivity. Through fitness and genomic analyses, we find that tripartite loops guide bacterial strains toward evolutionary paths that mitigate fitness costs and reverse resistance to component drugs in the loops and drive levels of resensitization not achievable through previously suggested pairwise regimens. We then apply this strategy to reproducibly resensitize or eradicate 4 drug-resistant clinical isolates over the course of 216 evolutionary experiments. Resensitization occurrs even when bacteria adapted through plasmid-bound mutations instead of chromosomal changes. Combined, these findings outline a sequential antibiotic regimen with high resensitization frequencies, which may improve the clinical longevity of existing antibiotics even in the face of antibiotic resistance.

## Introduction

Bacterial infections claim 7.7 million lives each year, of which 4.95 million are associated with antibiotic resistance (Murray et al. 2022). The slow pace of antibiotic development is failing to keep up with bacterial evolution, pushing us toward a postantibiotic era (Reardon 2014; Dutescu and Hillier 2021; Anon a). Tipping the scales in our favor in the fight against antibiotic resistance will require alternative strategies beyond the discovery or invention of new drugs to combat antibiotic resistance. One potential approach to slow down resistance evolution is to employ existing drugs in a sequence, with drugs administered one after the other at either predetermined times or as resistance arises (Andersson and Hughes 2010 ; Melnikov et al. 2020). Experimental and computational evolution studies indicate that sequential antibiotic regimens can constrain resistance evolution (Kim et al. 2014; Barbosa et al. 2019; Hernando-Amado et al. 2020; Aulin et al. 2021; Hernando-Amado et al. 2023; Nyhoegen and Uecker 2023), and incorporation of collateral sensitivity (CS) is thought to allow maintenance of sensitivity to the alternating drugs indefinitely (Hall et al. 2009; Imamovic and Sommer 2013; Baym et al. 2016; Barbosa et al. 2019).

Unfortunately, studies on the effectiveness, importance, and repeatability of CS have produced mixed results (Sakenova et al. 2025). Some experimental evolution studies report repeatable CS interactions (Podnecky et al. 2018; Barbosa et al. 2019; Hernando-Amado et al. 2023), while others show weak reproducibility (Podnecky et al. 2018; Maltas and Wood 2019; Nichol et al. 2019; Brepoels et al. 2022; Sørum et al. 2022). Reports also suggest that sequential antibiotic therapy can constrain resistance evolution independently of CS (Dunai et al. 2019; Brepoels et al. 2022). While the effect of CS on resistance evolution is anchored in several excellent studies which have identified a number of possible drug pairings, most pairings have been experimentally verified using a relatively limited number of evolutionary replicates (2 to 8 replicates in general; Imamovic and Sommer 2013; Barbosa et al. 2019; Hernando-Amado et al. 2020). Reproducibility is critical for the use of CS in the clinic, and the evolutionary tradeoffs that are at the core of sequential or cyclic regimens must be repeatable. Absent large scale experimental evolution studies it is unclear which drug cycles will fail, how often they will fail, and whether those failure rates can be limited.

In a previous study (Chowdhury and Findlay 2024), we showed that in a gentamicin (GEN)–piperacillin (PIP) pairwise cycle previously suggested for cyclic therapies (Imamovic and Sommer 2013; Barbosa et al. 2019), GEN resistant *Escherichia coli* lineages frequently evolved hypersensitivity toward PIP but subsequent PIP evolution failed to reverse resistance or reduce adaptation rates, predominantly producing multidrug-resistant mutants instead. The repeatability of CS evolution was low even in some previously reported CS-pairs, and there was a lack of complete antibiotic resensitization in most of the pairs tested (Chowdhury and Findlay 2024). This showed that CS interactions often fall apart due to lack of repeatability of evolution, and that pairwise cycles often do not achieve the level of resensitization required for cyclic regimens.

**Fig. 1.**
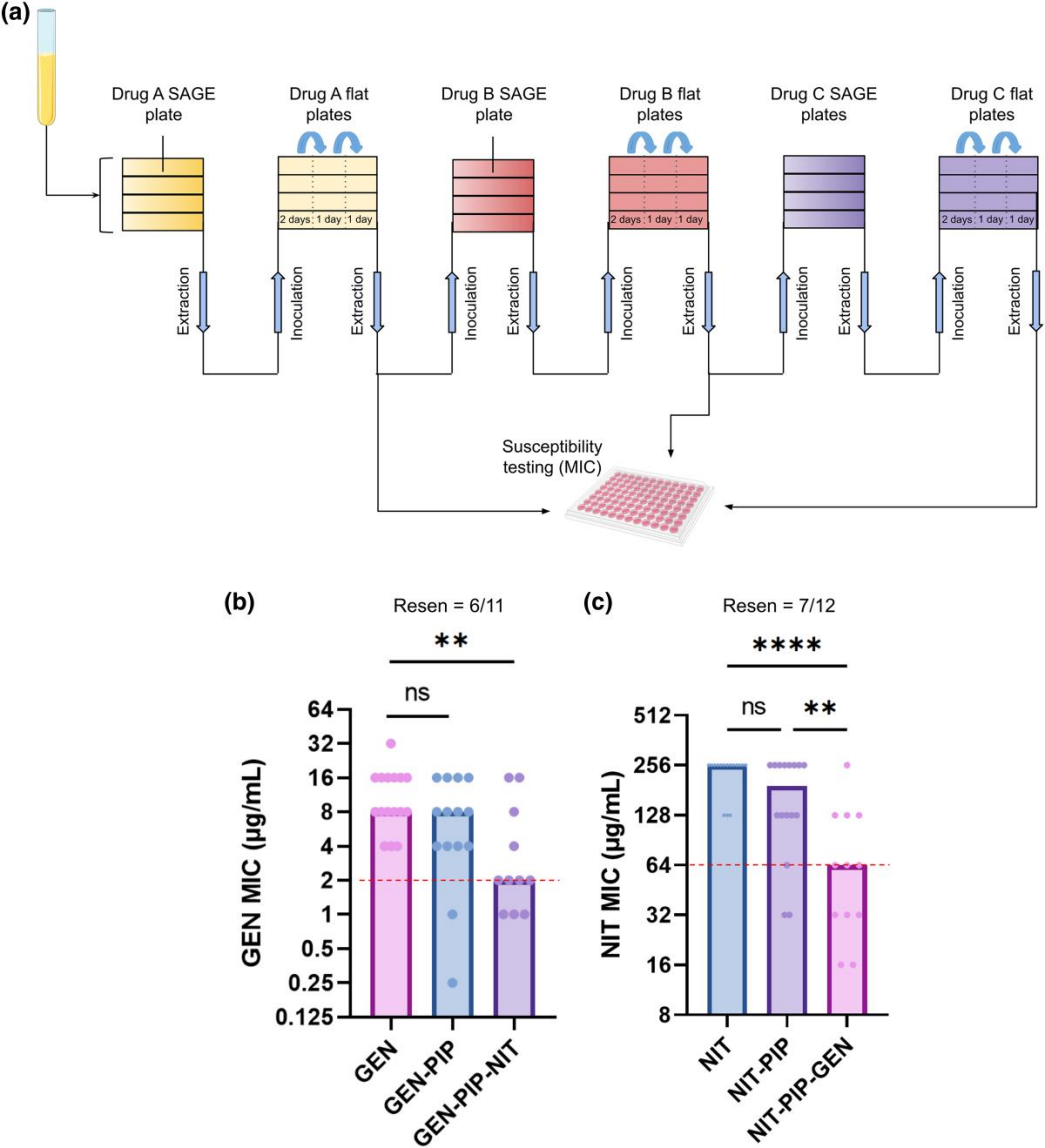
Tripartite loops improve antibiotic resensitization. a) SAGE is used to study 3-drug cyclic regimens or tripartite loops. Bacteria were inoculated into soft agar containing antibiotic gradients to generate resistant mutants (*n* = 16). SAGE plates were incubated for a fixed duration of 7 d, after which mutants were harvested and passed through 3 “flat plates” containing the same antibiotic from the prior SAGE plate at a concentration = ½ the evolved MIC of the mutants. The incubation period for each flat passage is noted in the figure. MIC and CS profiles of mutants were determined after the end of the flat plates. b) GEN MICs of strains that passed through the GEN-PIP-NIT tripartite loop. The *y* axis denotes the GEN MICs and the *x* axis denotes the sequence of antibiotics against which the strains were evolved. For example, the GEN-PIP bar shows the GEN MICs of strains that were sequentially evolved to GEN and PIP (as shown in a). c) NIT MICs of strains that passed through the NIT-PIP-GEN loop. Resen, resensitization counts. Dotted red lines indicate the clinical breakpoint (EUCAST). Bars represent the median MICs. **P < 0.01, ****P < 0.0001, Kruskal–Wallis with uncorrected Dunn's test.

In this study, we ask if mutants that fail to be resensitized in a pairwise cycle can be salvaged, with susceptibility to 1 or both of the initial antibiotics restored. Although resistant mutants possess strong selective advantages in environments containing the antibiotic of interest, those mutations render them less fit in antibiotic-free environments (Melnyk et al. 2015; Chowdhury and Findlay 2023). Reversion to susceptibility is then favored, either through competition by naive cells or by compensatory mutations that enhance fitness but lower resistance levels (phenotypic reversion; Allen et al. 2017; Dunai et al. 2019; Hernando-Amado et al. 2022). As it is infeasible to prescribe an antibiotic-free period during an ongoing infection, we instead incorporate a third antibiotic into the series, creating a tripartite loop (Fig. 1a). We choose this third drug with a mechanism of action distinct from the other 2, limiting the potential for cross resistance (Lázár et al. 2014; Lozano-Huntelman et al. 2020). We evolve replicates of *E. coli* K-12 substr. BW25113 (wildtype, WT; *n* = 16) through experimental tripartite loops using a soft agar gradient evolution (SAGE) based platform (Ghaddar et al. 2018). The large sample size allows us to capture repeatable evolutionary outcomes. Because compensatory evolution can frequently mitigate the effects of evolutionary tradeoffs (Sander et al. 2002; Ramadhan and Hegedus 2005; Goig et al. 2023; Eckartt et al. 2024), we include “flat plates” after every evolution step (Fig. 1a). Flat plates have been previously shown to reveal robust fitness tradeoffs (Chowdhury and Findlay 2023, 2024). Using this setup, we find that evolution of nitrofurantoin (NIT) resistance reliably restores GEN susceptibility in bacteria resistant to GEN and PIP when bacteria are evolved against drugs in the order GEN-PIP-NIT. This loop is effectively bidirectional, with NIT resistant bacteria reliably resensitized through a NIT-PIP-GEN loop. This effect is not limited to NIT, as a suboptimal drug like doxycycline (DOX), against which the majority of the GEN and PIP-resistant strains were cross-resistant, was able to reinstate GEN sensitivity. We find that to bypass the fitness loss associated with multidrug resistance, cells rewire their metabolic pathways, concurrently restoring susceptibility to the first drug in the series. All resensitizations we observe occur independently of CS interactions between component drugs in the loop. Extending our strategy to clinical strains, we then restore NIT sensitivity in clinical *E. coli* isolates that were initially resistant to NIT via sequential evolution against PIT (PIP/tazobactam) and GEN. Resensitization occurs even when bacteria bypass chromosomal PIP adaptations by mutating β-lactamases. Overall, we demonstrate that in some cases the multidrug resistance that arises in pairwise loops can be reversed by extending to tripartite loops, experimentally validating a path to more effective and more resilient cyclic antibiotic therapies.

## Results

### Tripartite Drug Loops that Resensitize Bacteria to Antibiotics

We previously reported using SAGE to generate 16 independent replicates of *Escherichia coli* K-12 substr. BW25113 (WT) resistant to both GEN and PIP (Chowdhury and Findlay 2024), a drug pair previously proposed to promote resensitization (Imamovic and Sommer 2013; Barbosa et al. 2019). Out of the 16 strains, 2 strains went extinct during PIP evolution, while the majority of the remaining 14 maintained resistance to GEN (Fig. 1b) (supplementary fig. S1a and f, Supplementary Material online) (Chowdhury and Findlay 2024). In this study, we screened for drugs that could resensitize these strains to GEN, extending our experimental design to incorporate evolution against a third drug “C” (Fig. 1a). We used SAGE to evolve resistance to antibiotics (Ghaddar et al. 2018), and after each stage of evolution, resistant lineages entered flat plates containing subinhibitory concentration of the challenge antibiotic (Fig. 1a). We incorporated flat plates into our experimental design to prioritize evolutionary tradeoffs that are less susceptible to compensatory evolution (Chowdhury and Findlay 2023). Fitness costs linked to resistance mutations are well-documented, but “cost-free” mutants frequently emerge in the clinic by offsetting these costs through compensatory mutations (Sander et al. 2002; Ramadhan and Hegedus 2005; Goig et al. 2023; Eckartt et al. 2024). If laboratory-generated resistance-associated tradeoffs can be readily alleviated, their therapeutic potential may be limited. We previously showed that flat plates can generate fitter mutants through compensatory evolution rapidly, ameliorating fitness deficits (Chowdhury and Findlay 2023). Any tradeoffs associated with the evolution of resistance in this study are therefore expected to be resilient against compensatory mutations.

Evolution of NIT resistance reduced the GEN resistance of 7 out of 11 strains to or below the clinical breakpoint, while driving 3 lineages extinct (supplementary fig. S1a, Supplementary Material online), with a median 8-fold drop in GEN MIC (Fig. 1b;  supplementary fig. S1f, Supplementary Material online). To account for possible random fluctuations in MIC measurements (Culp et al. 2020) affecting resensitization counts, we defined antibiotic resensitization as a 4-fold or greater reduction in MIC compared to levels evolved when they first encountered the antibiotic, in addition to reduction at or below the clinical breakpoint. Using this definition, NIT resistance resensitized 6 out of 11 strains to GEN (Fig. 1b).

To determine the effect of subsequent evolution against GEN, we subjected the 6 strains to GEN SAGE plates again, keeping the concentration of GEN equal to their first exposure. This is the second exposure of these strains to GEN: the first occurred during their initial evolution of resistance, and the second follows their resensitization to the drug. Although resensitized, the GEN MIC of these strains were 2- to 4-folds higher than the WT, making this a 2- to 4-fold smaller GEN challenge than the one faced by the WT (supplementary fig. S1f, Supplementary Material online). While we achieved a 100% evolution rate following the first GEN SAGE plate with WT bacteria (supplementary fig. S1a, Supplementary Material online) (Chowdhury and Findlay 2024), 3/6 lineages went extinct in this second exposure (supplementary fig. S1b, Supplementary Material online). This shows that not only were these strains resensitized to GEN, but their ability to develop GEN resistance was also impaired. The reinstatement of efficacy of GEN against initially GEN resistant strains therefore forms a 3-drug “loop.”

When we measured NIT resistance in the 3 surviving mutants, we observed a 4- to 16-fold reduction in NIT resistance levels, rendering all 3 strains resensitized to NIT (supplementary fig. S1d, Supplementary Material online). This hinted at a possibility of bidirectionality in this loop, where GEN and NIT resistance were mutually exclusive. To test this at scale, we restarted our evolution experiments with 16 replicates, this time evolving resistance sequentially to NIT, PIP, and then GEN. Following evolution against GEN we saw a ∼5-fold reduction in median NIT resistance (Fig. 1c). Nine out of 12 strains that completed this challenge fell at or below the NIT resistance breakpoint, with 7/12 reaching resensitization (Fig. 1c) (supplementary fig. S1g, Supplementary Material online). There were no extinctions on exposure to NIT or PIP, but 4 strains went extinct during GEN evolution (supplementary fig. S1c, Supplementary Material online).

When strains were evolved sequentially to PIP, GEN and NIT, NIT had no significant impact on PIP susceptibility (supplementary fig. S1e, Supplementary Material online). This showed that ordering of GEN, PIP and NIT was critical for achieving resensitization, but when applied correctly produced significant resensitizations.

### PIP Resistance is Important for Resensitization

**Fig. 2.**
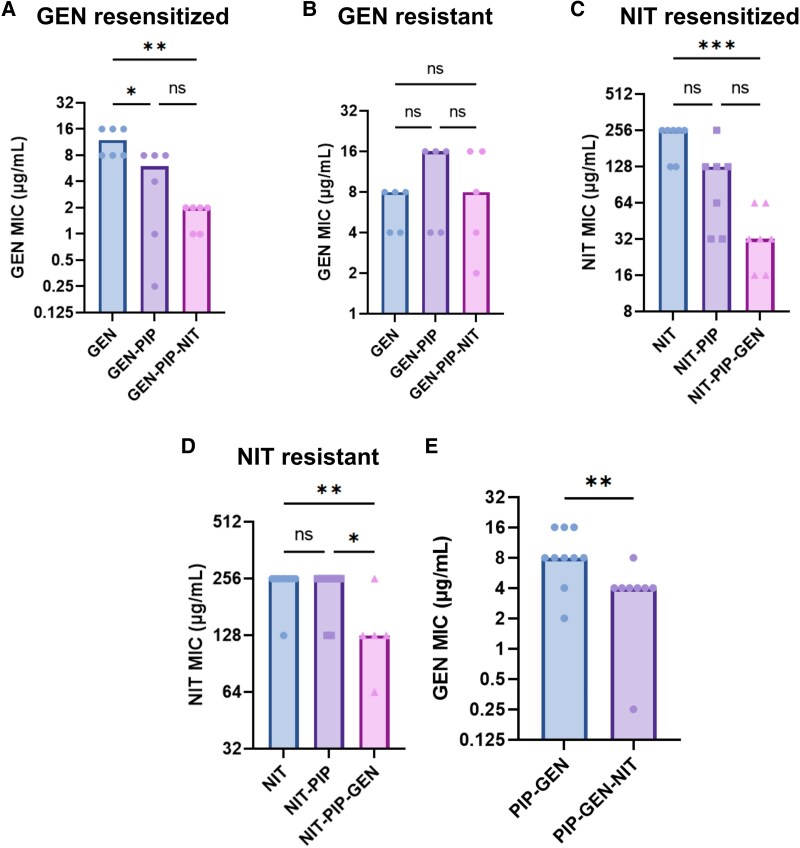
PIP aids resensitization in tripartite loops. a) GEN MICs of GEN-resensitized and b) GEN-resistant strains that passed through the GEN-PIP-NIT loop. c) NIT MICs of NIT resensitized and d) resistant strains that passed through the NIT-PIP-GEN loop. *P < 0.05, **P < 0.01, ***<*P* < 0.001, ****P < 0.0001, Kruskal–Wallis with uncorrected Dunn's test. e) GEN MIC of strains that passed through a PIP-GEN-NIT tripartite loop. MICs after the PIP step are not shown. For all graphs, the *y* axis denotes the MICs and the *x* axis denotes the sequence of antibiotics against which the strains were evolved before measuring the MICs. For example, the PIP-GEN-NIT bar shows the GEN MICs of strains that were sequentially evolved to PIP, GEN and NIT.**P < 0.01, Mann–Whitney test. Bars represent the median MICs.

Stratifying results from each step of the GEN-PIP-NIT loop by final GEN MIC revealed that strains which were ultimately resensitized to GEN exhibited decreased GEN resistance following PIP adaptation, while those that maintained GEN resistance after NIT were unchanged by evolution against PIP (Fig. 2a and b). Similarly, stratifying NIT resensitized and resistant strains from the NIT-PIP-GEN loop revealed that PIP evolution reduced NIT resistance by 2-fold in the resensitized strains, but not in the resistant ones (Fig. 2c and d). Overall, strains evolved through an intervening PIP evolution step exhibited a 4-fold reduction in GEN resistance on NIT exposure, as opposed to a 2-fold difference when the PIP step was omitted (Figs. 1b and 2e). This suggests that the incorporation of a third drug allows for resensitizations, which would not be possible in pairwise loops.

### Resensitizations are Independent of CS and Principally Mitigate Fitness Loss

To identify the driver of GEN resensitizations in the GEN-PIP-NIT loop, we first examined the effect of forward CS (Chowdhury and Findlay 2024) to NIT. To avoid missing even a weak connection between CS and resensitizations, we included all strains that showed any reduction in GEN resistance upon NIT evolution in the analysis.

**Fig. 3.**
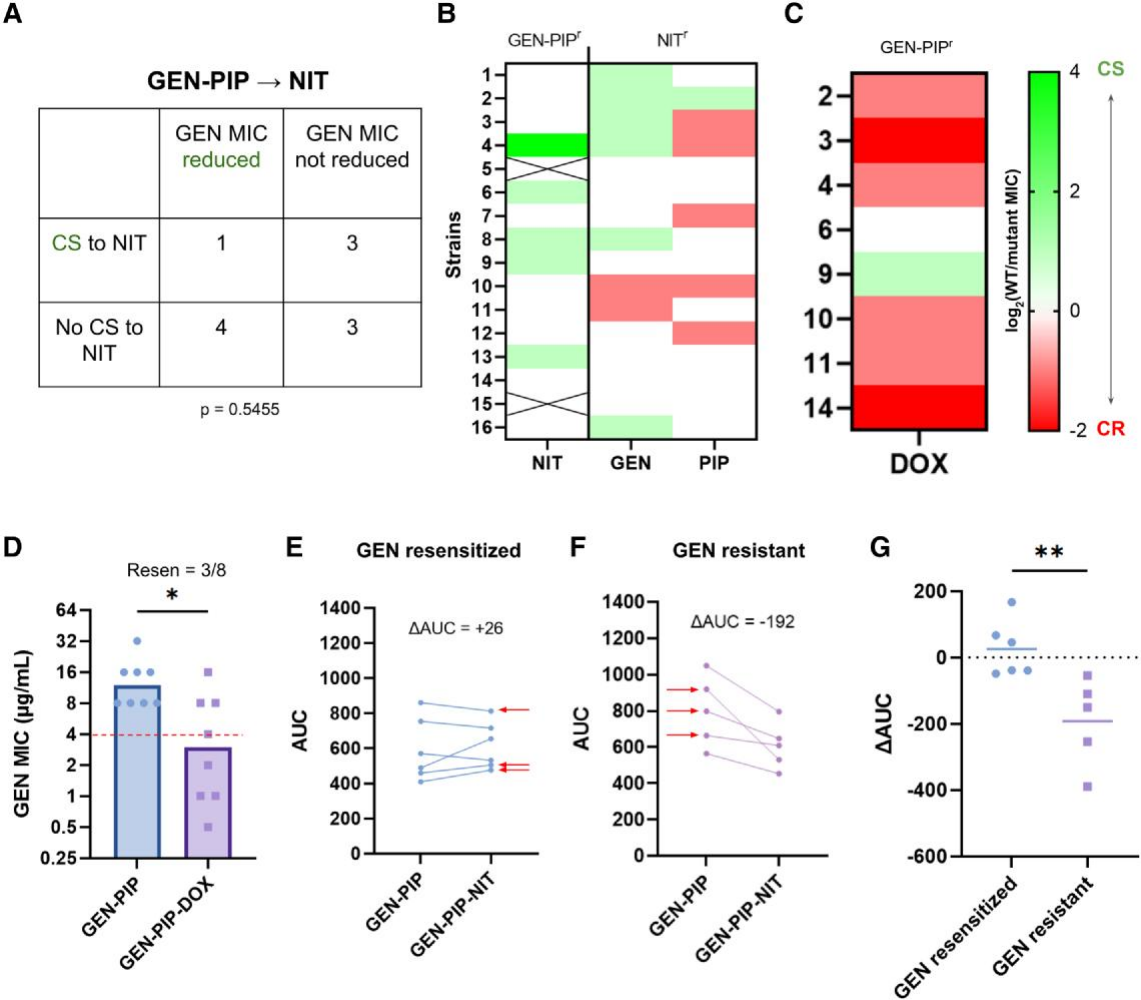
Resensitization does not correlate with CS but mitigates fitness loss. a) Contingency table for the 11 strains, which evolved NIT resistance through the GEN-PIP-NIT loop, showing no associations between CS and GEN resensitizations. Fisher's exact test. b) First column: NIT CS of the GEN and PIP evolved mutants from the GEN-PIP-NIT loop. Second column: GEN and PIP CS of WT bacteria evolved to NIT. CS interactions are reported on a log2 scale. c) DOX MICs of an 8 strain subset of the GEN and PIP evolved mutants from the GEN-PIP-NIT loop. CS interactions are reported on a log2 scale. The *y* axis denotes the ID of the strains that were picked for DOX MIC testing. d) GEN MICs of the subset that passed through the GEN-PIP-DOX loop. The *x* axis denotes the sequence of antibiotics against which the strains were evolved before measuring GEN MICs. For example, the GEN-PIP-DOX bar shows the GEN MICs of strains that were sequentially evolved to GEN, PIP, and DOX. Dotted line indicates the clinical breakpoint. Bars represent the median MICs. *P < 0.05, Mann–Whitney test. e and f) AUCs of strains before and after NIT evolution for GEN-resensitized and GEN-resistant strains, respectively. The *x* axis denotes the sequence of antibiotics against which the strains were evolved before measuring AUCs. GEN-PIP = before NIT evolution, GEN-PIP-NIT = after NIT evolution. ΔAUC is the average of the difference between post- and pre-NIT AUCs. For the GEN resistant group, we considered every strain that did not meet our resensitization criteria as resistant. This resulted in the inclusion of 1 strain that was below the GEN resistant breakpoint but did not reach our resensitization standard. Arrows indicate the strains that were sequenced. g) ΔAUC of individual strains plotted, grouped by resensitized and resistant. Horizontal lines represent the mean. **P < 0.01, unpaired *t*-test.

We found no correlation between NIT CS in the GEN and PIP multidrug-resistant strains and reductions in GEN resistance (Fig. 3a and b: left column; supplementary fig. S1f, Supplementary Material online: GEN MICs panel). To test if backward CS helped resensitize bacteria to GEN, we evolved 16 WT strains to NIT (flat plates included) and measured their GEN CS. The concept of forward and backward CS in sequential regimens was recently defined (Chowdhury and Findlay 2024). Briefly, in a sequential therapy transitioning from GEN to PIP, for example, if resistance to GEN leads to CS to PIP (i.e. CS from GEN to PIP), this is referred to as forward CS since the CS aligns with the direction of drug switching. If resistance to PIP results in CS to GEN (CS from PIP to GEN) and the sequence of drug application remains GEN to PIP, we describe this as backward CS as the CS runs opposite to the direction of evolution. When CS occurs in both directions, the drug pair is said to exhibit reciprocal CS (Kavanaugh et al. 2020). It is important to note that the designation of CS as forward or backward is always relative to the direction of drug switching.

Only 6/16 of these strains showed 2-fold CS to GEN (and just 1 with 2-fold PIP CS) (Fig. 3b; supplementary fig. S1g, Supplementary Material online: GEN MICs panel). In contrast, >50% of the strains were resensitized to GEN in the GEN-PIP-NIT loop, with a median 4-fold drop in resistance (Fig. 1b). This remained true for the NIT-PIP-GEN loop, with few CS interactions between the drugs (supplementary fig. S1g, Supplementary Material online). The resensitizations we observed appeared to be largely independent of forward CS, and while backward CS may have played a role, it was not strong enough to resensitize strains to the extent that we observed.

To test if the specific mechanism that conferred NIT resistance drove GEN resensitization, we evolved GEN-PIP multidrug-resistant lineages against DOX, a tetracycline antibiotic with a different mechanism of action from NIT, GEN, or PIP (Holmes and Charles 2009) (*n* = 8). Despite most of the 8 mutants showing cross-resistance to DOX (Fig. 3c), 5/8 strains dropped their GEN resistance to or below the resistance breakpoint, with 3/8 reaching resensitization (Fig. 3d). This provided further support that switching treatment to drugs toward, which bacteria exhibit CS is not required for resensitization, and indicated factors other than specific resistance pathways contributed to the resensitizations we observed.

Next, we hypothesized that the cumulative fitness costs of maintaining multiple drug resistance may promote the adoption of evolutionary paths that ameliorate these costs, resulting in phenotypic reversion. To test this, we measured strain fitness after each evolution step in the GEN-PIP-NIT, NIT-PIP-GEN, and PIP-GEN-NIT tripartite loops, using area under growth curves (AUC) as a proxy for fitness (supplementary fig. S2, Supplementary Material online) (Brepoels et al. 2022; Chowdhury and Findlay 2023). In the GEN-PIP-NIT loop, we found only a small drop in average fitness after each evolution step, which did not reach statistical significance (supplementary fig. S2a, Supplementary Material online). However, stratifying strains on the basis of resensitization to GEN revealed a clear difference in fitness (Fig. 3e-g). Strains that were resensitized to GEN upon NIT evolution either saw small gains or marginal losses in fitness (Fig. 3e and g), while those that retained GEN resistance lost significantly more fitness on average, with none gaining fitness (Fig. 3f and g).

The results of the NIT-PIP-GEN loop were less clear. We observed large fitness losses after every step of evolution, with the evolution of GEN resistance in particular producing extremely unfit mutants (supplementary fig. S2b, Supplementary Material online). Two of the 7 NIT resensitized strains exhibited moderate to large fitness gains upon GEN evolution but none of the 5 NIT resistant strains did (supplementary fig. S2d-f, Supplementary Material online). However, the differences in ΔAUC between the resensitized and resistant groups did not reach statistical significance (supplementary fig. S2f, Supplementary Material online).

Strains from the PIP-GEN-NIT loop showed a large fitness drop as they moved from PIP to GEN, but did not show a significant change in fitness following NIT evolution (supplementary fig. S2c, Supplementary Material online). Given the increased burden of resistance to 3 separate antibiotics, we expected that a constant AUC would correspond to significant resensitization. However, only 1 lineage exhibited increased PIP susceptibility, and that was following GEN evolution, not NIT (supplementary fig. S1e, Supplementary Material online). Looking more closely into the MIC profiles of these strains revealed that while there were no significant changes in PIP susceptibility, 7/8 strains had increased GEN susceptibility following NIT exposure (Fig. 3c). As GEN resistance was consistently associated with the largest fitness penalties (supplementary fig. S2, Supplementary Material online), this may have off-set a fitness penalty from acquiring NIT resistance, while leaving the less costly PIP resistance unchanged. Overall, the tripartite loops led to higher fitness costs and increased resensitization compared to pairwise loops.

### Whole Genome Sequencing Sheds Light on Resistance and Resensitization Mechanisms

**Fig. 4.**
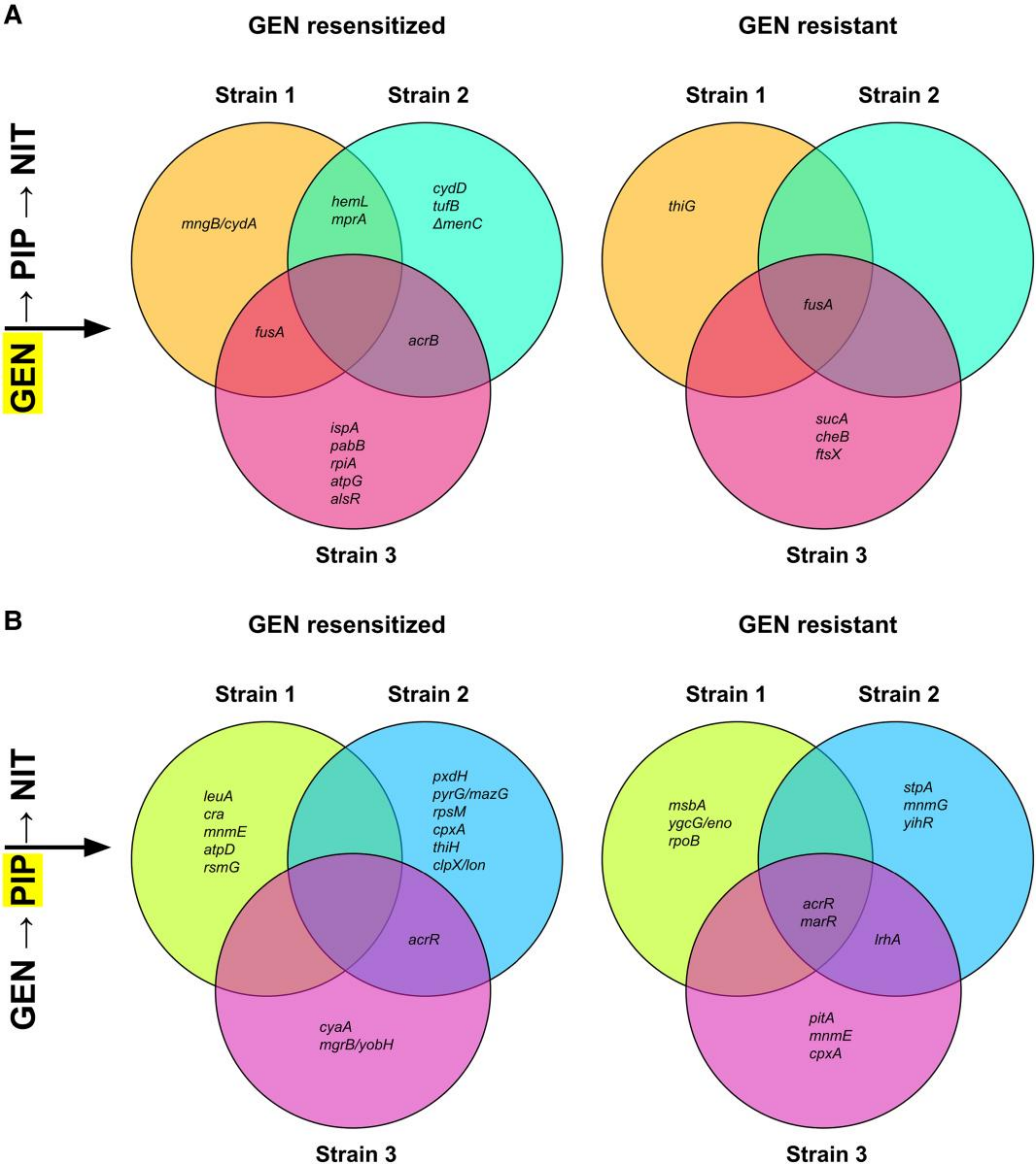

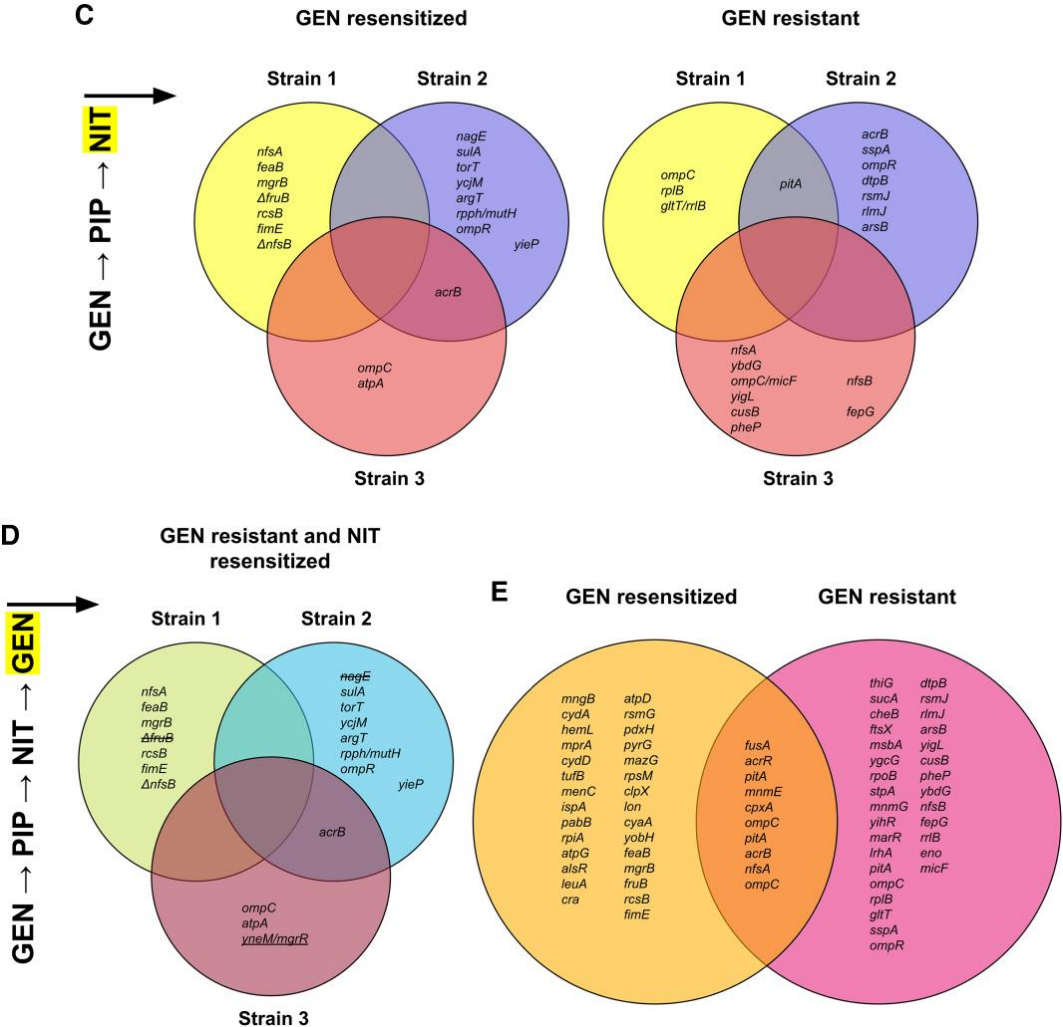

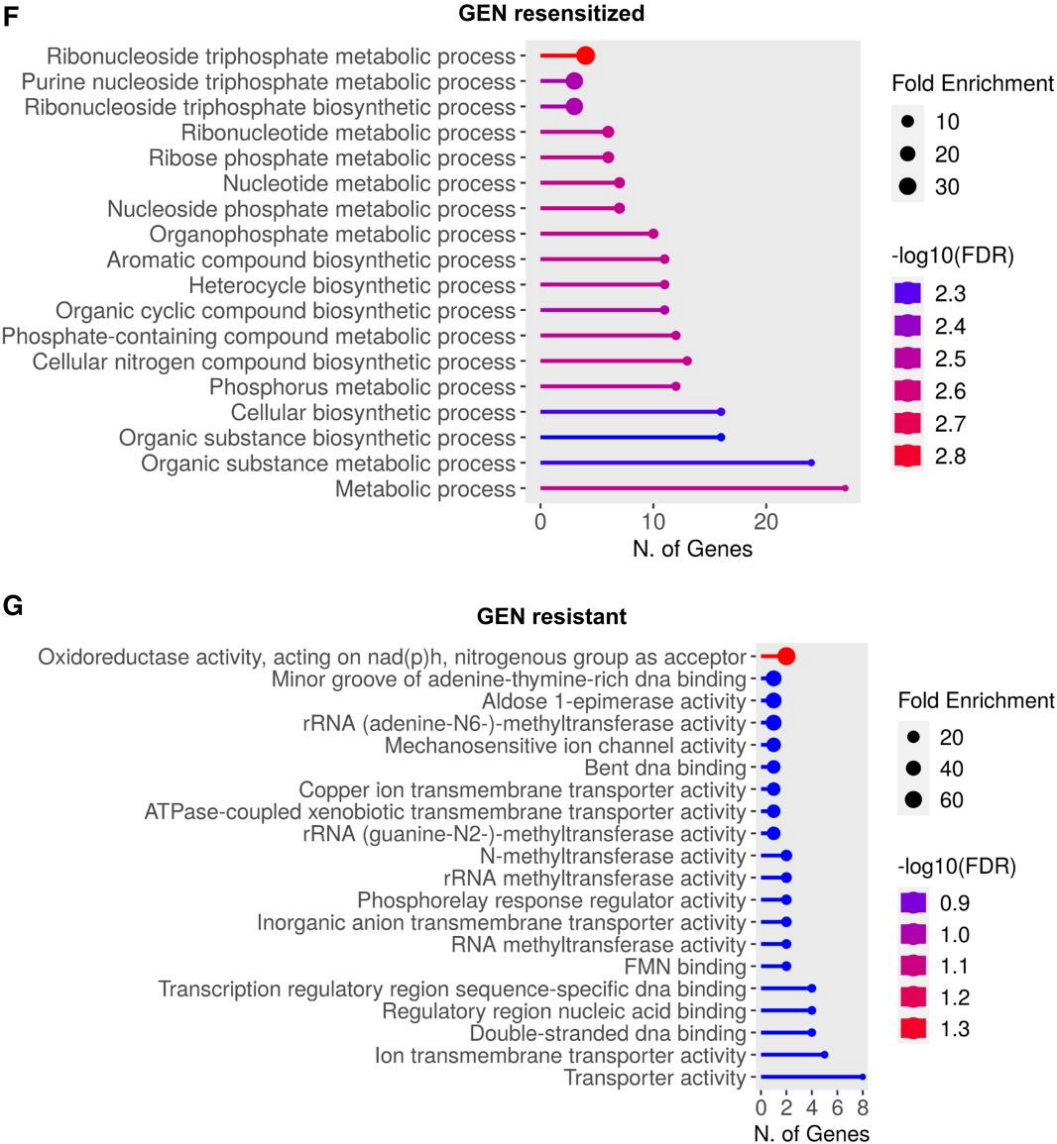
Tracking genomic changes through the GEN-PIP-NIT loop. a-d) Venn diagrams show overlapping and unique mutations in the GEN-resensitized and GEN-resistant strains from the 3 strains sequenced. The label on the left denotes when the strains were sequenced, with the most recent evolution step highlighted. Mutations that appeared in one step were carried forward to the next step, but are only displayed the first time they appeared in this figure. Strikethroughs denote mutations that appeared in a prior step but were not present in the current step. Underlined mutation in D, strain 3 represents a newly acquired mutation absent from strain 3 in (c). e) Venn diagram showing all overlapping and unique mutations between the GEN resensitized and GEN resistant group, pooled from every step (GEN-PIP-NIT only). f and g) GO term enrichment analysis of unique mutations in the GEN resensitized and GEN resistant groups.

To understand the genetic basis of the resistance and resensitization observed, we sequenced the genome of 6 lineages from the GEN-PIP-NIT loop: 3 that were resensitized to GEN and 3 that remained resistant to GEN after NIT evolution (supplementary fig. S1d, Supplementary Material online). Each lineage was sequenced following every evolution experiment (Fig. 1a), allowing us to reconstruct all 6 evolutionary trajectories (Fig. 4a-d).

Five of the 6 lineages acquired their initial GEN resistance through mutations in the translation elongation factor G, *fusA*, mutations that are known to reduce GEN's ability to bind to the ribosome (Fig. 4a) (Rodriguez de Evgrafov et al. 2020). Even though both the GEN resensitized and resistant groups evolved similar GEN MICs (supplementary fig. S1f, Supplementary Material online), the resensitized strains contained multiple additional mutations in genes involved in the electron transport: *hemL* (Choby and Skaar 2016), *cydA* (Cotter et al. 1997), *cydD* (Poole et al. 1994), *menC* (Kurosu and Begari 2010), and *atpG* (Ofori-Anyinam et al. 2020) (Fig. 4a). Mutations in the electron transport chain can provide GEN resistance either by disrupting drug uptake or reducing ribosomal protein levels (Shan et al. 2015; Shiraliyev and Orman 2024).

Five out of the 6 lineages acquired mutations in the efflux regulators *acrR* and *marR* following exposure to PIP, changes known to confer β-lactam resistance (Fig. 4b) (Dulanto Chiang and Dekker 2024). Mutations in the resensitized group also included other genes involved in β-lactam resistance such as *cpxA* and *cyaA* (Jing et al. 2021; Gross et al. 2024); genes involved in carbon, amino acid, and vitamin metabolism: *cra* (Shimada et al. 2005), *leuA* (Somers et al. 1973), *pdxH*, and *thiH* (di Salvo et al. 2002; Kriek et al. 2007); and the ribosomal genes *rsmG* and *rpsM* (Hoang et al. 2004; Okamoto et al. 2007) (Fig. 4b). The resistant group did not show any clear mutations in genes involved in metabolism or the ribosome.

All NIT-resistant mutants acquired mutations in 1 or more of the genes involved in NIT resistance: *nfsA, nfsB* (Dulyayangkul et al. 2024), *sulA* (essential for NIT resistance in *lon* mutants) (Roemhild and Andersson 2021), *ompR* (Le et al. 2019), and *ompC* (Mohakud et al. 2023; Hussein et al. 2024) (Fig. 4a). Both GEN-resensitized and GEN-resistant lineages showed multiple mutations involved in transmembrane transporters. The resensitized group acquired mutations in genes involved in the sugar phosphotransferase transport system: *fruB* (Reizer et al. 1995) and *nagE* (Peri and Waygood 1988), which also have putative roles in aminoglycoside uptake (Lang et al. 2023), while the resistant strains gained mutations in metal ion, amino acid and peptide transporters instead: *cusB* (Outten et al. 2001), *fepG* (Chenault and Earhart 1992), *pitA* (Beard et al. 2000), *dptB* (Harder et al. 2008), and *pheP* (Cosgriff et al. 2000) (Fig. 4a). A GEN uptake assay suggested that these transport related mutations in the GEN resensitized strains may have slightly increased GEN penetration, but the results did not reach statistical significance (supplementary fig. S3, Supplementary Material online).

To elucidate how differences between the GEN-resensitized and GEN-resistant groups could affect their propensity toward resensitization, we first identified overlapping and unique mutations between the 2 groups following NIT evolution (Fig. 4e). Common mutations were mostly those known to confer resistance to GEN, PIP, or NIT, as discussed above. To categorize the remainder, we ran GO term enrichment analyses on the non-overlapping gene sets. Every hit from the resensitized group that was above the enrichment FDR cutoff was involved in metabolic processes (Fig. 4f); whereas no significant enrichment was found in the resistant group. Manually removing the FDR cutoff (by setting it to 0.99) identified processes involved in transport and DNA-binding (Fig. 4g). Mutations in metabolic processes are often involved in compensatory evolution to mitigate fitness costs and phenotypic reversion of resistance (Durão et al. 2015; Richardson et al. 2015; Fondi et al. 2016; Zampieri et al. 2017), which supports our observation of the little to no loss (but rather a slight increase) in fitness in the GEN resensitized strains (Fig. 3c), in contrast to the significant fitness loss in the resistant group (Fig. 3d). These genomic and fitness outcomes suggest that cells become resistant to antibiotics using similar mechanisms, but bifurcate at the level of fitness cost compensation. Cells that adopt pathways that help mitigate their fitness losses also reverse their resistance to the earlier drugs, strongly suggesting a correlation between the 2 phenotypes.

Since we also saw a surprising drop in NIT resistance after reacquisition of GEN resistance from the GEN-PIP-NIT-GEN series in all 3 non-extinct lineages (supplementary fig. S1d, Supplementary Material online), we looked at the genome sequence of these NIT resensitized strains (Fig. 4d). After reacquisition of GEN resistance, the genomic profile of the 3 strains looked almost identical (Fig. 4c and d) except that strain 1 was replaced by a mutant with an intact *fruB* gene possibly via elevation of a low frequency mutant in the population, while strain 2 reverted its *nagE* mutation (Fig. 4d). Both genes are involved in sugar transport. It is unclear how reversion of these mutations allowed GEN resistance reacquisition. There are no direct reports of NIT being transported inside the cell via these transporters, but both *nagE* and *fruB* have been reported to carry other drugs like streptozotocin and fosfomycin (Lengeler 1980; Gil-Gil et al. 2021). Since the *nagE* and *fruB* mutations are the only differences between the GEN-sensitive-NIT-resistant and GEN-resistant-NIT-sensitive strains (Fig. 4c and d), it is likely that these mutations play a role in GEN and/or NIT resistance levels.

### NIT-PIP-GEN Loop Reduces Clinically Acquired NIT Resistance

**Fig. 5.**
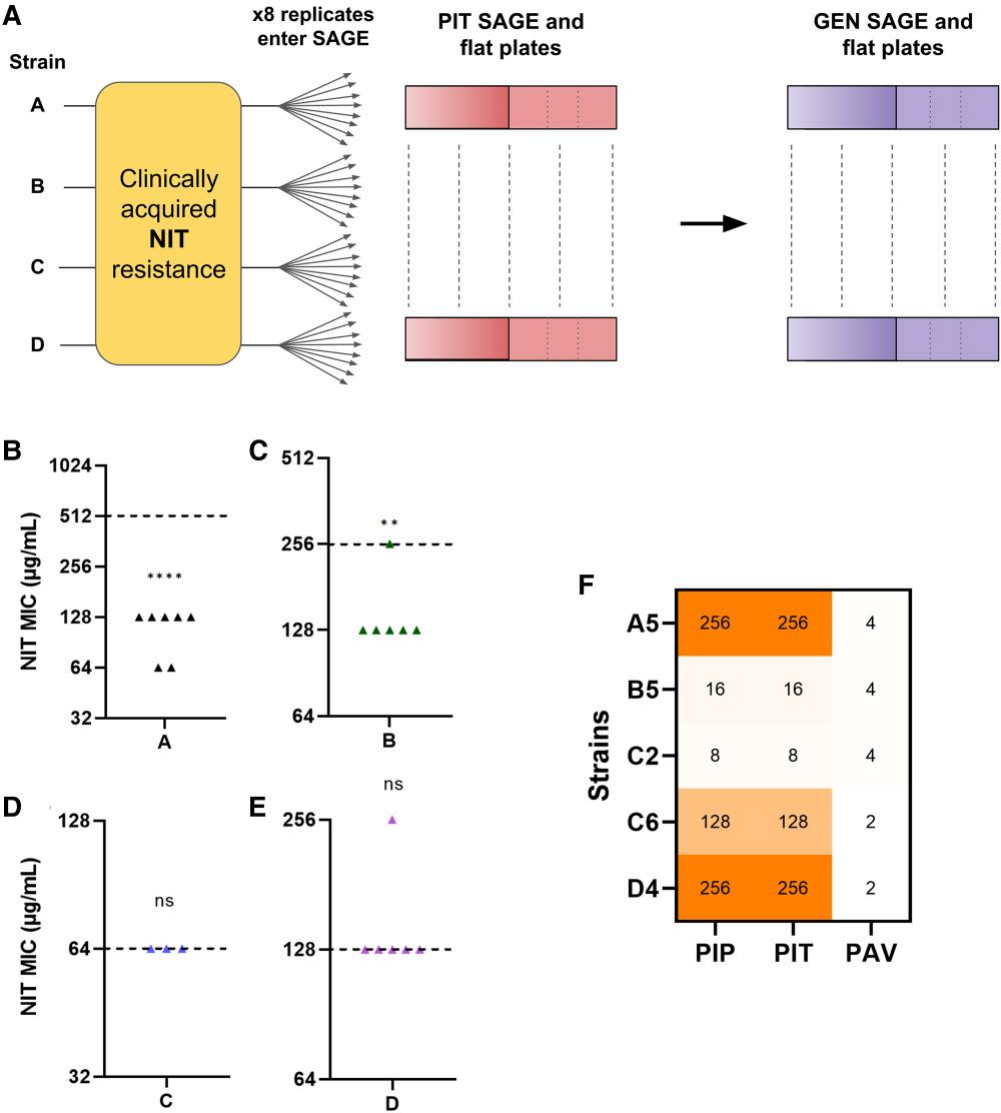
The NIT-PIP-GEN loop reduces clinically acquired NIT resistance. a) Four uropathogenic clinical *E. coli* strains resistant to NIT were used to start sequential PIT and GEN evolution. Each strain started 8 replicates in SAGE. The rest of the experimental evolution design remained identical to the one used for the laboratory strain. b–e) NIT MICs of the clinical replicates post-GEN adaptation. The dotted line represents NIT MICs of the parental strain pre-SAGE adaptation. Labels on the *x* axis denote the parental strain of the replicates for which the MICs are displayed. **P < 0.01, ****P < 0.0001, 1 sample *t*-test. f) PIP, PIP and PAV MICs of 5 clinical replicates after PIT exposure. The *y* axis denotes the strain ID where A5 = the 5th replicate from strain A.

To test if a tripartite loop can reduce clinically acquired drug resistance, we obtained 4 previously reported NIT-resistant uropathogenic *E. coli* clinical isolates (Bielec et al. 2023): strains A, B, C, and D (renamed for this study) (Fig. 5a). The strains were all resistant to NIT at varying levels (Fig. 5b-e), and were isolated using sampling criteria designed to avoid repeated collection of the same isolates (Bielec et al. 2023). Next, we started sequential SAGE evolutions with 8 replicates for each strain (Fig. 5a). Three of the 4 strains (A, C, and D) were confirmed to have β-lactamase(s) via MIC testing (PIP MIC > 64 μg/mL, PIP/tazobactam MIC ≤ 4/4 μg/mL), so we opted to replace the PIP SAGE plates with PIT (PIP/tazobactam) plates, which contained the same PIP concentrations used in the rest of study in combination with a flat tazobactam concentration of 4 μg/mL throughout the plate (Ambrose et al. 2003). Subsequent GEN SAGE plates remained the same. These strains showed an extinction pattern similar to our NIT-PIP-GEN evolutions using the laboratory strain, with PIT evolution not incurring any extinctions and GEN evolution causing a ∼31% extinction (10/32 strains extinct) comparable to the 25% with the laboratory strain (supplementary figs. S1c and S4a, Supplementary Material online).

Post-GEN evolution, strain A showed a 4-fold median reduction in NIT resistance with 5/8 replicates showing MICs of 128 μg/mL, 2 dropping to 64 μg/mL (below the NIT clinical breakpoint), and 1 going extinct (Fig. 5b) (supplementary fig. S4a, Supplementary Material online). Strain B showed a 2-fold median reduction in NIT resistance, with 5/8 strains dropping to 128 μg/mL down from 256 μg/mL of the parent strain and 2 going extinct (Fig. 5c) (supplementary fig. S4a, Supplementary Material online). 5/8 replicates of strain C went extinct (supplementary fig. S4a, Supplementary Material online), and the rest of the replicates did not show a significant drop in NIT resistance, and neither did the 6 surviving replicates of strain D (Fig. 5d and e).

### Bypassing Chromosomal Adaptations Against PIP Does Not Abolish Resensitizations

When we measured PIT MICs of the clinical replicates after PIT SAGE plates, we noticed resistance levels that were 8- to 16-folds higher (supplementary fig. S4b, Supplementary Material online) than the PIP resistance levels we observed after PIP SAGE plates using our laboratory strain (supplementary fig. S1f and g, Supplementary Material online). These high resistance levels were limited to Strains A and D, and to a smaller extent C, which were also the strains that contained at least 1 β-lactamase that inactivated PIP. All 8 replicates of Strain B, which was originally sensitive to PIP (and hence did not require tazobactam for PIP activity) evolved an MIC of 16 μg/mL, similar to the laboratory strain. This led us to hypothesize that instead of chromosomal adaptations against PIP during the PIT exposure, the β-lactamase bearing strains may have mutated their β-lactamase to resist tazobactam instead.

We picked 5 of these PIT resistant strains and measured their PIP MICs, but this time in combination with the non-β-lactam β-lactamase inhibitor avibactam (at a flat concentration of 4 μg/mL) (PAV) (Nichols et al. 2018). Addition of avibactam increased PIP sensitivity by 64- to 128-folds, returning MICs to within 2-fold of the PIP MICs of the parental strain (Fig. 5f) (supplementary fig. S4b, Supplementary Material online), indicating that the PIP resistance was mediated by a change in the β-lactamase that allowed cells to bypass inhibition by tazobactam. Replicate B5 also saw a small 4-fold drop in PIP resistance which may be due to the modest antibacterial activity of avibactam against *E. coli* (Berkhout et al. 2015). Despite avoiding chromosomal adaptations to PIP, we still observed almost identical extinction frequencies in all 4 clinical strains (supplementary figs. S1c and S4a, Supplementary Material online) and frequent and significant resistance drops in strains A and B (Fig. 5b and c).

## Discussion

Cyclic antibiotic therapies have been proposed as a way to combat the rise of antibiotic resistance (Kim et al. 2014; Barbosa et al. 2019 ; Hernando-Amado et al. 2020, 2023; Aulin et al. 2021). The success of such regimens is thought to hinge on CS interactions between the component drugs (Hall et al. 2009; Imamovic and Sommer 2013; Baym et al. 2016; Barbosa et al. 2019). However, to date, CS has seen no application in the clinic since its first description in 1952 (Szybalski and Bryson 1952) and since its proposed benefits in cyclic therapies, partly because of the unreliability and rarity of CS (Podnecky et al. 2018; Maltas and Wood 2019; Nichol et al. 2019; Sørum et al. 2022). In a previous study, we showed that CS, when applied in the correct direction during cyclic therapies, can help resensitize bacteria to antibiotics (Chowdhury and Findlay 2024). However, we observed that the repeatability of CS evolution was low even in drug pairs with reported CS interactions, typified by small resistance drops and low resensitization frequencies, which may readily lead to the emergence of multidrug-resistant mutants (Chowdhury and Findlay 2024).

In this study, we explored the potential of extending pairwise regimens to longer “tripartite loops.” We found that the tripartite loop GEN-PIP-NIT significantly improved resensitization frequencies as compared to the previously proposed GEN-PIP pairwise loop (Imamovic and Sommer 2013; Barbosa et al. 2019; Chowdhury and Findlay 2024), going from ∼14% (Chowdhury and Findlay 2024) to ∼54% of lineages (Fig. 1b). The loop was also invertible, with NIT-PIP-GEN reliably resensitizing bacteria to NIT (Fig. 1c). Resensitizations were independent of CS (Fig. 3a and b) (supplementary fig. S1f and g, Supplementary Material online), and did not appear to be driven by NIT-specific resistance mutations. The resensitization was at least partially independent of drug identity, as extending the GEN-PIP loop with DOX, against which the bacteria showed cross-resistance, produced GEN-resensitized strains (Fig. 3c and d).

When GEN-resensitized strains from the GEN-PIP-NIT loop were subjected to GEN again, we found that the antibiotic posed a significant evolutionary challenge, with 3 out of the 6 strains going extinct during SAGE (supplementary fig. S1b, Supplementary Material online). We did not observe extinctions when WT bacteria were exposed to GEN (supplementary fig. S1a, Supplementary Material online), implying that multidrug-resistant bacteria have constrained evolutionary paths that limit further resistance development. In fact, the 3 mutants that were able to reacquire GEN resistance dropped their NIT resistance in the process, showcasing the difficulty in maintaining multiple resistance mechanisms.

Unlike in the laboratory, rapid drug cycling in patients may not be possible due to pharmacokinetic factors (Nyhoegen and Uecker 2023), and the resulting longer evolutionary periods can allow for compensatory evolution, which can mitigate evolutionary tradeoffs like CS (Durão et al. 2018; Sørum et al. 2022; Sakenova et al. 2025). While this complicates CS-based cyclic therapies, our study shows that compensatory evolution can be leveraged to drive phenotypic reversion of resistance. We tracked fitness of resensitized and resistant bacteria throughout our tripartite loops, demonstrating that the sequential adaptation to 3 antibiotics increased fitness penalties compared to pairwise loops (supplementary fig. S2, Supplementary Material online), possibly due to the need to carry multiple independent resistance phenotypes. Strains could overcome this fitness loss through resensitization, e.g. to GEN (Figs. 3e and 4), or could persist with poor growth (Fig. 3f) (Durão et al. 2018). This interplay was also apparent in the PIP-GEN-NIT loop, again through resensitization of GEN (Fig. 3d). Since GEN evolution imposed the largest penalties in our experiments, it appears to be ideal for incorporation into drug cycling protocols. Our GO term enrichment analyses also clearly show evidence of metabolic rewiring associated with compensatory evolution (Durão et al. 2015; Richardson et al. 2015; Fondi et al. 2016; Zampieri et al. 2017) in resensitized strains that are missing from the resistant ones (Fig. 4f and g), and the fitness and genomic analyses taken together suggests a strong association between compensatory evolution and resistance reversion.

In further support of longer cyclic regimens, we showed that despite the fact that PIP evolution failed to produce significant resensitizations (Fig. 1b and c) (supplementary fig. S1f and g, Supplementary Material online) (Chowdhury and Findlay 2024), it aided in bringing down resistance to the initial drug in both the GEN-PIP-NIT loop and NIT-PIP-GEN loops (Fig. 2a-d), which turned to full resensitizations after evolution against the last drug in the series. Additionally, tripartite loops continued to drive bacterial extinction (supplementary fig. S1a-c, Supplementary Material online), reinforcing prior work on sequential regimens (Barbosa et al. 2019; Chowdhury and Findlay 2024).

When we compared the NIT resistant versus NIT resensitized strains from the GEN-PIP-NIT-GEN sequence (Fig. 4d), we discovered that the genomes of the 2 groups were almost identical, except that the NIT resensitized strains reinstated 2 sugar transporter mutations. Elucidation of the exact mechanism of NIT resensitization will require further studies, but our data suggests a possible, previously unreported role for sugar transporters in NIT resistance (Fig. 4c and d).

Our results from the evolutions using uropathogenic clinical strains show that our suggested tripartite loops may be effective even against diverse genetic backgrounds and when resistance evolution is complicated via plasmid-bound evolution, showing potential for translation into the clinic. Overall, we suggest that tripartite loops can improve antibiotic resensitization and allow continuation of antibiotic cycling even if pairwise cycles fail, without being limited by CS requirements. With our antibiotic development pipeline failing to keep up with resistance emergence, such cyclic therapies may prolong the lifespan of our existing antibiotics.

## Materials and Methods

### Bacterial Strain and Growth Conditions

*E. coli* K-12 substr. BW25113 and the evolved lineages were cultured in Muller Hinton (MH) media at 37 °C. Antibiotics were added to the growth media as needed to grow or isolate resistant mutants from SAGE plates. The clinical samples were streaked on tryptic soy agar plates containing 64 μg/mL of NIT, and pure cultures were obtained by transferring a single colony from each strain onto MH agar plates. These were then used for all subsequent experiments.

### SAGE Evolutions

Evolutions were performed as previously described (Chowdhury and Findlay 2024; Chowdhury et al. 2025). SAGE evolved mutants were extracted from within 1.5 cm of the end of the plates after 7 days of incubation into MH broth containing the challenge antibiotic at a concentration = 2 × the WT MIC. Strains were considered extinct when they could not be recovered after extraction from within 1.5 cm of the end of the SAGE plates (Chowdhury and Findlay 2024). Mutants that went extinct were given a second chance at evolution using the same parameters as before. This allowed us to maintain a larger sample size through the extinction events that occurred at different steps of evolution, and we report both the initial and final extinction counts (supplementary fig. S1a-c, Supplementary Material online). Antibiotic concentrations in SAGE are listed below, and were determined from trial SAGE experiments to reliably evolve strains with MICs above the clinical breakpoints for each antibiotic (Anon).

**Table:** 

Antibiotic	Concentration (μg/mL)
GEN	5
PIP	40
NIT	80

### Susceptibility Testing

Minimum inhibitory concentrations (MIC) of antibiotics were determined using the broth microdilution method as described by the CLSI (2018). Antibiotics were diluted in MH broth, and then serially diluted across 96 well plates. Bacteria were inoculated at a concentration of 1/200 of a 0.5 McFarland standard. Plates were incubated overnight at 37 °C without shaking, and the MIC was recorded as the lowest antibiotic concentration that prevented visible bacterial growth. For PIT and PAV MICs, tazobactam or avibactam respectively was added at a fixed concentration of 4 μg/mL to all the wells in the test plates (Ambrose et al. 2003; Nichols et al. 2018).

### Flat Plates

Flat plates were prepared as previously described (Chowdhury and Findlay 2024). First, the evolved MIC of the antibiotic used in the preceding SAGE plates was determined for all strains that completed SAGE evolution. Next, specific lanes were created for each strain by pouring ∼12 mL medium supplemented with the antibiotic at half the MIC of that strain into 4-well dishes. This allowed for the maintenance of the resistance gained from SAGE during compensatory evolution. Each replicate underwent 3 consecutive passages on these flat plates (Fig. 1a). The first plate was incubated for 2 d, and the second and third plates for 1 d (Fig. 1a). Unlike during SAGE evolutions, where extractions were limited to within the final 1.5 cm of the plates, cells from flat plates were extracted from the farthest point of growth. PIT flat plates contained tazobactam at a fixed concentration of 4 μg/mL in combination with appropriate [PIP] (Ambrose et al. 2003).

### Fitness Measurements

Growth curves for each strain were made by tracking absorbance readings at 595 nm of 1/100 dilutions of overnight cultures using a plate reader (Tecan Sunrise) for 24 h. Plate lids were treated with 0.05% Triton X-100 in 20% ethanol to reduce fogging (Brewster 2003). AUCs were calculated using GraphPad Prism.

### Whole Genome Sequencing

Genomic DNA was extracted using the Bio Basic genomic DNA kit (Cat. no.: BS624). Sequencing and variant calling were performed by Seqcenter (USA) on an Illumina NextSeq 2000, and demultiplexing, quality control, and adapter trimming were performed with bcl-convert (v3.9.3). Variant calling was performed using Breseq under default settings (Barrick et al. 2014). NCBI reference sequence CP009273.1 was used for variant calling. Common mutations were identified using custom R scripts and Venn diagrams were based on the output of the R package *ggvenn*.

### Term Enrichment Analysis

To identify pathways affected by the mutations observed, ShinyGO v0.81 (https://bioinformatics.sdstate.edu/go/) was used. For GEN resensitized strains, the following parameters were used: Species: *E. coli* str. K-12 substr. MG1655 STRINGdb; DB: Go Biological processes; FDR: Default of 0.05. Resistant strains produced no result using these parameters. These parameters were modified by removing the FDR cutoff to produce the results shown in Fig. 4g. The modified parameters were: DB: GO Molecular Function; FDR: set to 0.99.

### GEN Uptake Assay

GEN uptake was measured using a modified version of a previously reported protocol (Chen et al. 2019). 300 μL of overnight bacterial cultures were transferred into 30 mL of MH broth in conical flasks and incubated at 37 °C with 250 rpm shaking until log phase was reached. The log phase of each strain was estimated from their growth curves. OD at 600 nm was then measured for each strain, and cells were either concentrated or diluted to reach an OD of 0.4. 100 μL of cells were transferred into microcentrifuge tubes, and GEN was added at a concentration of 100 μg/mL. Tubes were allowed to incubate at 37 °C with 1,000 rpm shaking on a heat block for 15 min. Tubes were then chilled on ice for 5 min, then centrifuged at 12,000 × *g* for 2 min. 5 μL of the supernatants were used to spot WT *E. coli* seeded MH agar plates, and left to air dry. Plates were incubated overnight, then photographed from a fixed distance of 29 cm. Images were analyzed by fitting circles around the inhibition zones and measuring the area in px^2^ using ImageJ (Schneider et al. 2012). Measurements were taken from 6 independent replicates for each strain. The 3 resensitized strains that were sequenced (supplementary fig. S1f, Supplementary Material online) were also used to perform this test.

## Supplementary Material

msaf115_Supplementary_Data

## Data Availability

All genomic data have been deposited in the NCBI Sequence Read Archive (SRA) under BioProject ID: PRJNA1254677.
