# Supplementary material for: Tripartite Loops Reverse Antibiotic Resistance
Source: Mol Biol Evol. 2025 Jun 6;42(6):msaf115. doi: 10.1093/molbev/msaf115 (PMC12164588; doi:10.1093/molbev/msaf115)
Supplement: msaf115_Supplementary_Data [file msaf115_supplementary_data.pdf]

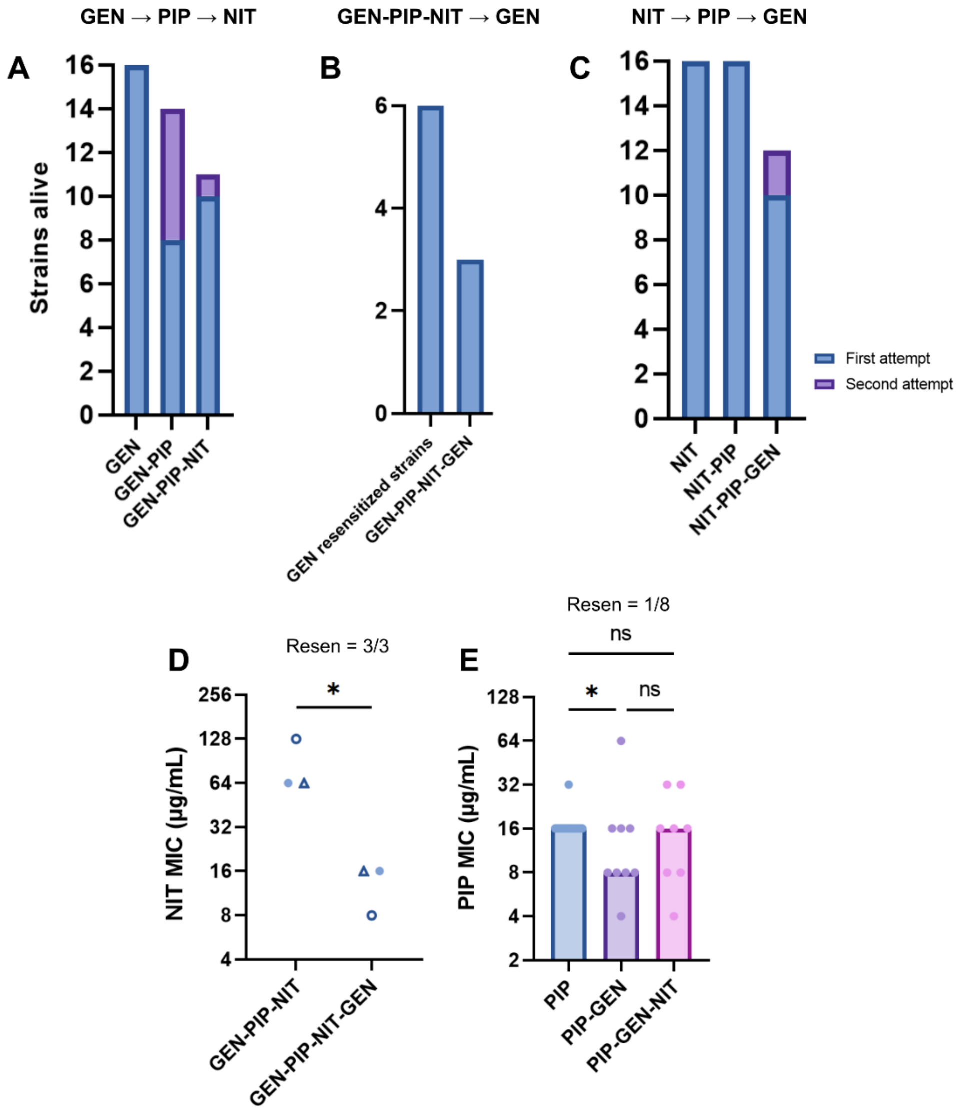
GEN → PIP → NIT
GEN-PIP-NIT → GEN
NIT → PIP → GEN
A
B
C
Strains alive
GEN
GEN-PIP
GEN-PIP-NIT
GEN resensitized strains
GEN-PIP-NIT-GEN
NIT
NIT-PIP
NIT-PIP-GEN
First attempt
Second attempt
D
E
Resen = 3/3
Resen = 1/8
*
ns
NIT MIC (µg/mL)
PIP MIC (µg/mL)
PIP
PIP-GEN
PIP-GEN-NIT

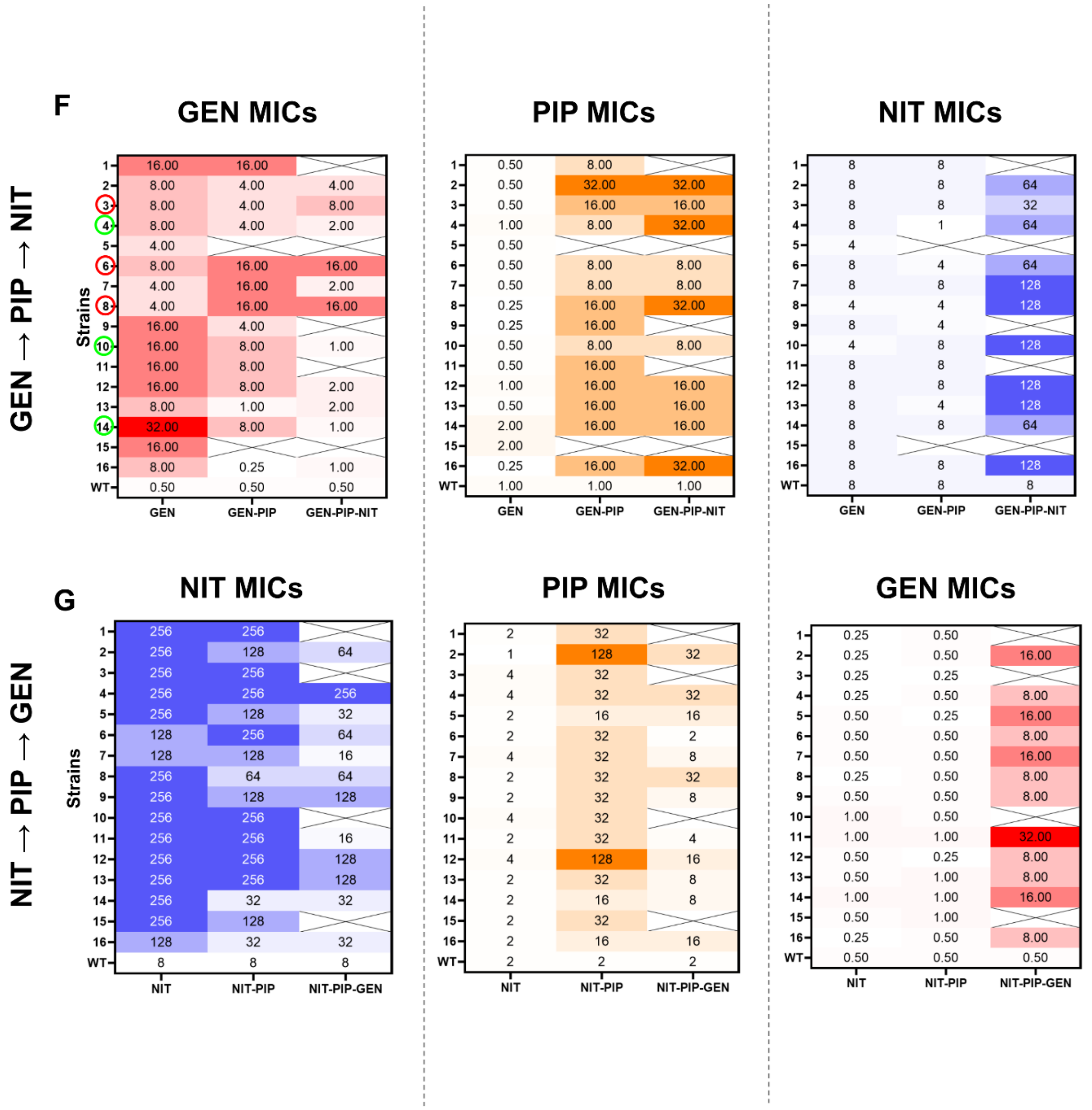


Fig. S1: (A) Number of non-extinct strains following each step of the GEN-PIP-NIT loop. (B) Non-extinct strains following the GEN-PIP-NIT-GEN loop. Only strains resensitized to GEN after teh GEN-PIP-NIT loop are included in the analysis. (C) Non-extinct strains following each step of the NIT-PIP-GEN loop. Purple stacked bars denote strains that went extinct on the initial pass, but survived a second attempt. (D) NIT MICs of the three non-extinct after the GEN-PIP-NIT-GEN sequence. *p<0.05, unpaired t-test (E) PIP MICs of strains that passed through the PIP-GEN-NIT loop. Bars represent the median MICs. *p<0.05, Kruskal-Wallis with uncorrected Dunn's test. (F) and (G) MICs of every strain evolved in the GEN-PIP-NIT and NIT-PIP-GEN loops. Green and red circles indicate the sequenced GEN-resensitized and GEN-resistant strains that were sequenced, respectively. The x-axes indicate the drugs against which which bacteria were evolved, with the MIC antibiotic listed at the top of each panel.

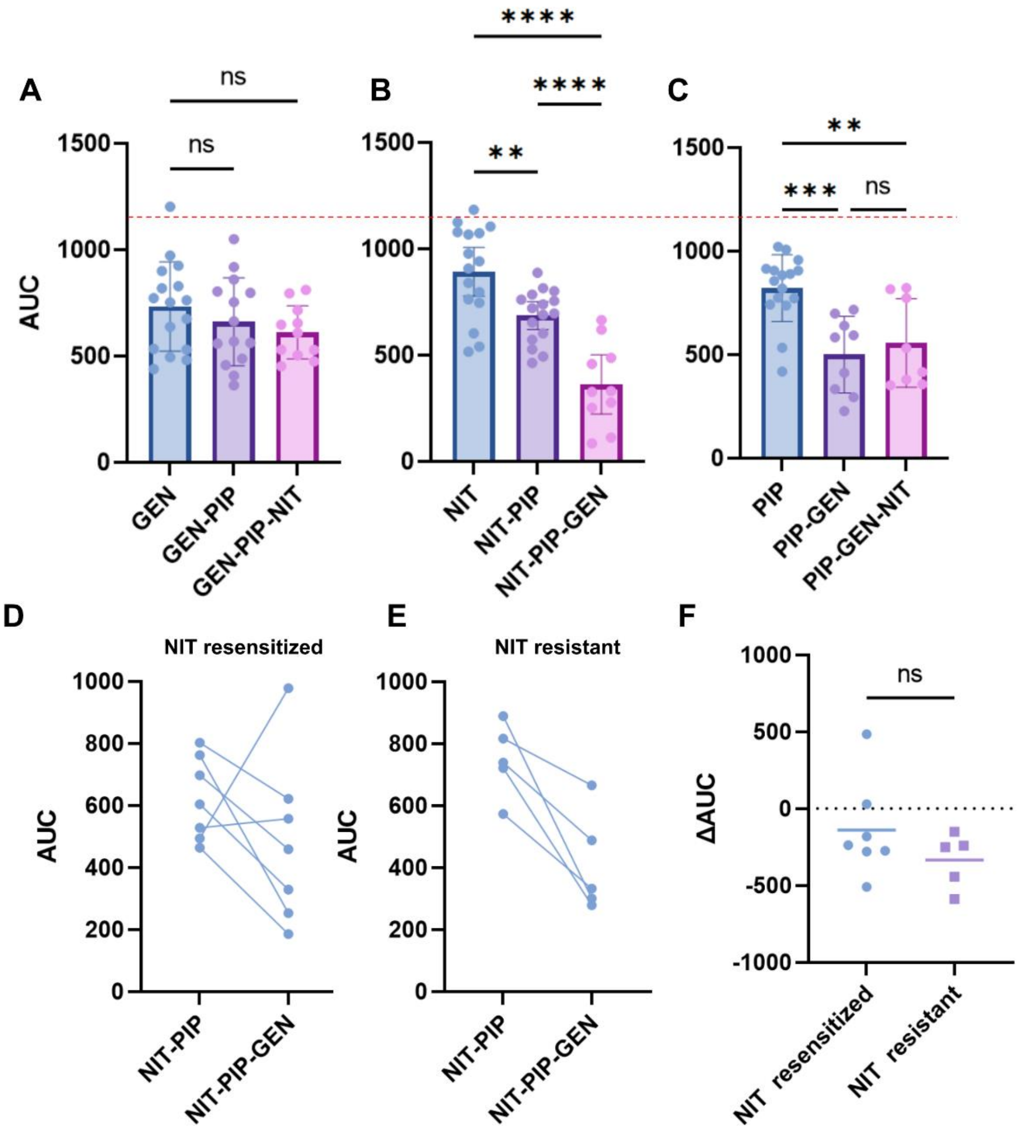


Fig. S2: AUC data of strains passed through (A) GEN-PIP-NIT, (B) NIT-PIP-GEN, and (C) PIP-GEN-NIT loops. The red dotted line denotes the fitness of the WT strain. Bars represent the mean with 95% CI. *p<0.05, **p<0.01, ***p<0.001, ****p<0.0001, one-way ANOVA with Fisher's LSD test. (D) and (E) AUCs of strains before and after GEN evolution for NIT-resensitized and NIT-resistant strains, respectively. The x-axes denotes the sequence of antibiotics against which the strains were evolved before measuring AUCs. (F) ΔAUC of individual strains plotted, grouped by resensitized and resistant; unpaired t test used to test significance. Means indicated by horizontal lines.

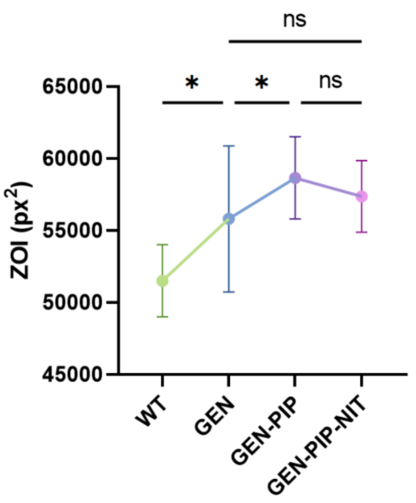


Fig. S3: Results from the gentamicin uptake assay. GEN was incubated with bacteria (n = 3), and then centrifuged to pellet the cells. The supernatant was used to spot E. coli seeded plates (more details in Materials and Methods). The lower the GEN uptake, the more GEN remaining in the supernatant after centrifugation and hence, the larger the ZOI. Error bars represent SD. *$p<0.05$ one-way ANOVA with Fisher's LSD test.

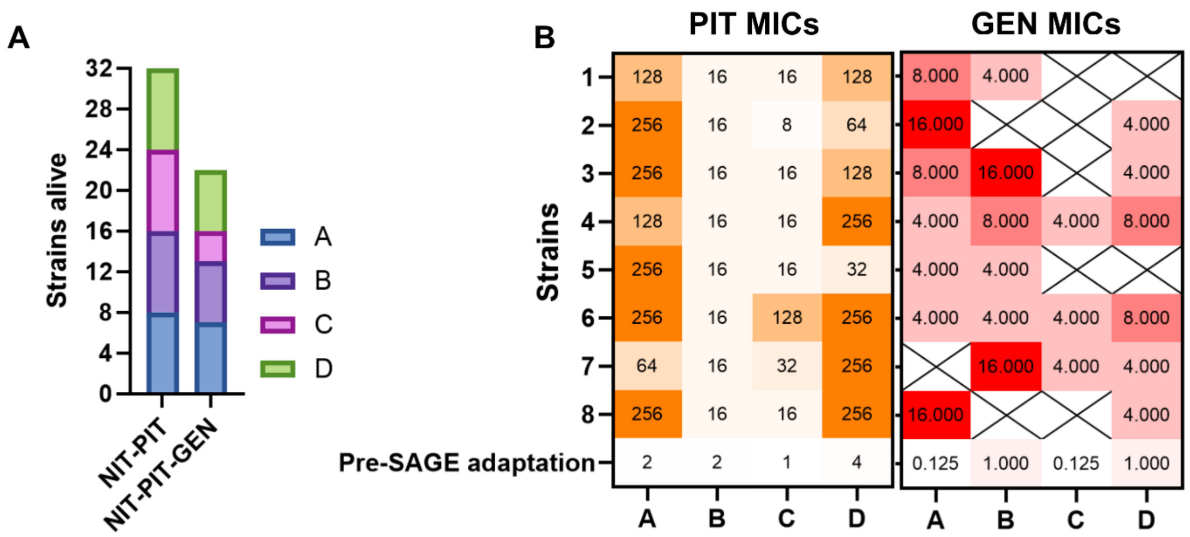


Fig. S4: (A) Number of non-extinct strains following each step of the (NIT)-PIT-GEN loop. NIT is omitted since the clinical strains were already NIT resistant. The different colored bars represent strain A, B, C or D. (B) PIT and GEN MICs of the clinical replicates following PIT and GEN adaptation respectively.
